# Robust Adaptive Beamforming with Optimal Covariance Matrix Estimation in the Presence of Gain-Phase Errors

**DOI:** 10.3390/s20102930

**Published:** 2020-05-21

**Authors:** Di Yao, Xin Zhang, Bin Hu, Qiang Yang, Xiaochuan Wu

**Affiliations:** 1 School of Information Science and Engineering, Harbin Institute of Technology at Weihai, Weihai 264209, China; yaodi@hit.edu.cn; 2 Electronic and Information Engineering, Harbin Institute of Technology, Harbin 150001, China; zhangxinhit@hit.edu.cn (X.Z.); 15B905008@hit.edu.cn (B.H.); yq@hit.edu.cn (Q.Y.)

**Keywords:** robust adaptive beamformer, INC matrix reconstruction, sensor gain-phase errors, compressed sensing

## Abstract

An adaptive beamformer is sensitive to model mismatch, especially when the desired signal exists in the training samples. Focusing on the problem, this paper proposed a novel adaptive beamformer based on the interference-plus-noise covariance (INC) matrix reconstruction method, which is robust with gain-phase errors for uniform or sparse linear array. In this beamformer, the INC matrix is reconstructed by the estimated steering vector (SV) and the corresponding individual powers of the interference signals, as well as noise power. Firstly, a gain-phase errors model of the sensors is deduced based on the first-order Taylor series expansion. Secondly, sensor gain-phase errors, the directions of the interferences, and the desired signal can be accurately estimated by using an alternating descent method. Thirdly, the interferences and noise powers are estimated by solving a quadratic optimization problem. To reduce the computational complexity, we derive the closed-form solutions of the second and third steps with compressive sensing and total least squares methods. Simulation results and measured data demonstrate that the performance of the proposed beamformer is always close to the optimum, and outperforms other tested methods in the case of gain-phase errors.

## 1. Introduction

Adaptive beamforming, which can enhance the signal of interest (SOI) while suppressing interferences automatically, is a data-dependent method and is widely applied in the fields of array signal processing, including radar, sonar, and wireless communication, etc. [1,2,3,4]. The standard Capon beamforming is one of the well-known types, which can achieve the optimum output signal-to-interference-plus-noise ratio (SINR) under ideal conditions without any model mismatches. Yet in a practical radar system, various non-ideal factors are inevitable to exist, such as sensor gain-phase errors, the sample covariance matrix contains the SOI and sensor failure (referred to as “sparse array”), etc. Therefore, in the past decades, various robust adaptive beamforming (RAB) methods have been proposed for improving the robustness of the Capon beamformer, which can be divided into the following two major types:

The first type is non-reconstruction approaches, directly using the sample covariance matrix (SCM) to calculate the adaptive weights, including the diagonal loading (DL) algorithms [5,6], the shrinkage algorithms [7], the worst-case-based (WCB) algorithms [8], and the eigenspace-based (ESB) algorithms [9,10]. Among them, the DL algorithms are one of the most widely used robust techniques by adding a diagonal parameter matrix to the sample covariance matrix, while the optimal DL factors are hard to determine in different scenarios. The WCB algorithms can avoid the SOI self-cancellation with a constraint, but the output performance is very sensitive to the accuracy of presumed prior knowledge of the steering vector (SV). Unfortunately, the required uncertainty of estimation is hardly satisfied in the actual system. The ESB algorithms are proposed to reduce the SV mismatches of the SOI, via projecting the desired SV into the signal-plus-interference subspace of the SCM. However, it will suffer severe performance degradation at a low signal-to-noise ratio (SNR). In [11], a new beamformer design problem is studied with limited prior knowledge, which is very robust for the pointing errors.

The second type is INC matrix reconstruction approaches, which can completely eliminate the SOI from the SCM and is first proposed by Gu [12]. In [12], the INC matrix is reconstructed by integrating over the complement sector of the SOI angular sector, while the SOI SV is estimated by solving a quadratically constrained quadratic programming problem. On this basis, Shen proposed two novel types of beamformers based on the INC matrix reconstruction and the SV estimation of the SOI [13,14]. They can acquire excellent robustness in the case of existing pointing errors and limited snapshots. By exploiting the sparsity of the signals in the whole angle sector, a low-complexity reconstruction method is proposed in [15]. It effectively reduces the computational complexity while taking into account the robustness of the algorithm. In [16], the INC matrix is reconstructed by combining a *p*-shrinkage operator and alternating direction method, which can achieve good performance with only single snapshot. 

Nevertheless, the above methods are under the assumption that the array model is ideal. In a practical system, the array model is often unpredictable in advance because of the existing sensor gain and phase errors, which could significantly degrade the beamformer performance. In [17,18], adaptive beamforming methods are proposed with the self-calibration technique of sensors gain-phase errors, but they are very sensitive to the SV mismatches when SNR is high. In [19], an expanded robust capon beamformer is presented, which improves the robustness against the array model errors. However, the method suffers serious performance degradation when the errors are large. Recently, a self-calibration RAB algorithm is proposed in [20], which allows the INC matrix to be reconstructed effectively when sensor gain-phase errors are calibrated beforehand. Unfortunately, it is only suitable for a uniform linear array (ULA) and requires that the partial sensors errors are accurately known.

In this paper, a novel adaptive beamformer with broad robustness is proposed to overcome the previous works limitations. The beamformer can be simultaneously suitable for the problems of the ULA, the sparse array, the gain-phase errors of the sensors, the training samples containing the SOI and the pointing errors. The major contribution of this work is presented in the following three aspects. To eliminate the influence of the gain and phase errors, an error model of the array SV is first formulated with the sparse representation of the signal and first-order Taylor series expansion. Then, the calibration coefficients, the SVs of the SOI and interferences can be jointly estimated using an alternating descent method. Meanwhile, the Gerschgorin disks estimation method is employed to obtain the number of signals. After array calibration, a quadratic optimization problem is established to accurately solve the interferences power coefficients. In order to reduce the computational complexity, the closed-form solution is derived with compressed sensing (CS) and total least squares (TLS) methods. Finally, the INC matrix can be reconstructed as a linear combination of the products of the interference SVs and the corresponding powers. The trials of simulation and the real data measured by high-frequency surface wave radar (HFSWR) are designed to express the effectiveness and superiority of the proposed algorithm. 

The rest of this paper is organized as follows. The sparse signal model and some necessary background are given in Section 2. Section 3 outlines the proposed gain and phase errors model of the sensors and the corresponding self-calibration method, the estimation method of sources number, SVs, and powers of the SOI and interference, and the sparse reconstruction algorithm of the INC matrix. In Section 4, numerical simulations are presented. The verification of the measured data is designed in Section 5. The conclusions are drawn in Section 6.

## 2. Signal Model

### 2.1. Sparse Signal Model

Considering a uniform linear array composed of M sensors with inter-element spacing d. Let K+1 far-field narrow-band signals (one SOI and *K* interferences) impinge on the ULA. The array observation vector at the *t*-th snapshot can be formed as:

(1) $$x\left(t\right)=\sum_{k=0}^{K}a\left(\theta_{k}\right)s_{k}\left(t\right)+n\left(t\right)=A\left(\theta \right)S\left(t\right)+n\left(t\right)\;t=1,2,\cdots ,T,$$

where $A\left(\theta \right)=\left[a(\theta_{0}),a(\theta_{1}),\cdots ,a(\theta_{K})\right]$ is the array manifold matrix, $S\left(t\right)={\left[s_{0}\left(t\right),s_{1}\left(t\right),\cdots ,s_{K}\left(t\right)\right]}^{T}$ denotes the corresponding signal complex envelops, n(t) is the additive white Gaussian noise, *T* indicates the number of sampling snapshots. $a\left(\theta_{k}\right)$ is the ideal SV at the direction $\theta_{k}$, which can be expressed as:

(2) $$a\left(\theta_{k}\right)={\left[1,e^{-j\frac{2\pi d}{\lambda }\sin(\theta_{k})},\cdots ,e^{-j(M-1)\frac{2\pi d}{\lambda }\sin(\theta_{k})}\right]}^{T},$$

where λ denotes the signal wavelength. Assuming the situation that the number of signals is less than the number of sensors. The signals can be considered to be sparse when the directions of signals only occupy certain isolated points in the observation angles. Let Q indicates the number of the grid, all the signals are on the grids. Suppose the entire angle space covers from −90° to 90°, and is divided into Q equal grids with 1° spacing. Now, the new array manifold matrix (called as dictionary) A, and signal complex envelops can be respectively redefined as:

(3) $$A=\left[a(\theta_{1}),a(\theta_{2}),\cdots ,a(\theta_{Q})\right]$$

(4) $$S\left(t\right)={\left[0_{1},0_{2},\cdots ,s_{0}\left(t\right),\cdots ,0,s_{K}\left(t\right),\cdots 0_{Q}\right]}^{T}.$$

It is clear that signal sparsity is satisfied because few non-zero value exists in the S(t). The observation vector can be rewritten as x(t)=AS(t)+n(t). When the receiving array is sparse, that is, only D(D<M) array elements can be used to sample the space signals, other elements are invalid. Considering the failure elements are randomly distributed in the receiving array, now, the sparse observation vector can be obtained by setting the corresponding rows to zero from the observation vector of the full array.

### 2.2. Adaptive Beamforming

The output of the adaptive beamformer is defined as:

(5) $$y\left(t\right)=w^{H}x\left(t\right),$$

where w is the adaptive weight vector. The optimal weight vector w can be obtained based on the maximum output signal-interference-noise-ratio criterion, which is mathematically equivalent to the following optimization problem:

(6) $$\min_{w}\;w^{H}R_{i+n}w\;\mathrm{subject}\;\mathrm{to}w^{H}a\left(\theta_{0}\right)=1,$$

where H denotes Hermitian transpose, $R_{i+n}$ is the ideal INC matrix, $\theta_{0}$ indicates the true direction of the SOI. The optimal solution of the above problem can be written as:

(7) $$w=\frac{R_{i+n}^{-1}a\left(\theta_{0}\right)}{a{\left(\theta_{0}\right)}^{H}R_{i+n}a\left(\theta_{0}\right)}.$$

In the practice, since the true direction $\theta_{0}$ of the SOI and the ideal INC matrix $R_{i+n}$ are unavailable, they are generally replaced by the nominal direction and the SCM $\hat{R}=\sum_{t=1}^{T}x\left(t\right)x^{H}\left(t\right)/T$ respectively. As T→∞, the SCM will converge to the ideal INC matrix. However, when the number of sampling snapshots T is small, there is a large difference between $R_{i+n}$ and $\hat{R}$. At this time, the beamformer performance will be severely degraded when the SCM contains the SOI or there is an SV mismatch problem. Besides, [15] shows that the gain-phase errors of the sensors can further aggravate the mismatch problem.

## 3. Proposed Algorithm

### 3.1. Gain and Phase Error Model

In the presence of gain and phase errors, the predefined dictionary cannot effectively represent the exact array manifold, which will seriously degrade estimation performance of signal SVs. In this section, we focus on establishing a model of the gain and phase errors. Considering the gain and phase uncertainties are spatially invariant and time-invariant, now, the observation vector can be defined as:

(8) z(t)=GAS(t)+n(t)

(9) $$G=\mathrm{diag}\left\{\delta g_{1}e^{j\delta \varphi_{1}},\delta g_{2}e^{j\delta \varphi_{2}},\cdots ,\delta g_{M}e^{j\delta \varphi_{M}}\right\},$$

where diag{·} indicates the diagonal matrix, $\delta g_{m}$ and $\delta \varphi_{m}$ denote the gain and phase errors at *m*-th sensor, respectively. Let the first sensor be the reference sensor, that is, $\delta g_{1}=1$ and $\delta \varphi_{1}=0$.

In order to simultaneously estimate the true directions of the signals and gain-phase errors, as stated in [17], we convert the SVs with the errors into a sum of the true SVs and gain-phase errors. Based on the first-order Taylor series expansion, the transformation expression can be shown as:

(10) $$Ga(\theta_{k})=a(\theta_{k})+\mathrm{diag}\left\{a(\theta_{k})\right\}\Delta ,$$

where $\mathrm{diag}\left\{a(\theta_{k})\right\}\Delta$ expresses the error term, Δ is an *M*-dimensional complex vector that can be given by:

(11) $$\Delta_{m}=\delta g_{m}+j\delta \varphi_{m}+j\delta g_{m}\delta \varphi_{m}m=1,2,\cdots ,M.$$

Thus, after conversion, the observation vector can be rewritten as:

(12) $$z\left(t\right)=AS\left(t\right)+\left[\Delta^{1},\Delta^{2},\cdots ,\Delta^{Q}\right]⊙AS\left(t\right)+n\left(t\right)=\left(A+P\right)S\left(t\right)+n\left(t\right).$$

Because the gain and phase errors independent of directions of the signals, thus, we can obtain:

(13) $$\Delta^{1}=\Delta^{2}=\cdots =\Delta^{Q}=\Delta ,$$

where ⊙ is the Schur–Hadamard product. It is noticed that the true signal SVs, gain and phase errors can be estimated when the P and S(t) are precisely solved.

### 3.2. The Estimation of the Gain-Phase Errors and Signal SVs

#### 3.2.1. Gain Errors Estimation

The SCM can be computed with the *T* data snapshots with $\hat{R}=\sum_{t=1}^{T}z\left(t\right)z^{H}\left(t\right)/T$, which can be eigen-decomposed as:

(14) $$\hat{R}=\sum_{m=1}^{M}\gamma_{m}u_{m}u_{m}^{H},$$

where $\gamma_{m}$ indicates the eigenvalues arranged in descending order, $u_{m}$ denotes the corresponding eigenvector. The largest K+1 eigenvalues ${\left\{\gamma_{m}\right\}}_{m=1}^{K+1}$ are signal eigenvalues, the others ${\left\{\gamma_{m}\right\}}_{m=K+2}^{M}$ are noise eigenvalues.

Then, the estimated noise power can be given as:

(15) $${\tilde{\sigma }}_{n}^{2}=\frac{1}{M-K-1}\sum_{m=K+2}^{M}\gamma_{m}.$$

Define $\hat{R}\left(m,m\right)$ as the *m*-th diagonal element of $\hat{R}$. Gain errors can be obtained by [21]:

(16) $$\delta g_{m}=\sqrt{\frac{\hat{R}\left(m,m\right)-{\tilde{\sigma }}_{n}^{2}}{\hat{R}\left(1,1\right)-{\tilde{\sigma }}_{n}^{2}}}m=1,2,\cdots ,M.$$

#### 3.2.2. The Estimation of Phase Errors and Signal SVs

After calibrating the gain errors, the phase errors and signal SVs can be acquired by solving the sparsity regularized problem [22]:

(17) $$\underset{S\left(t\right),P}{\min}{\left\|P\right\|}_{F}^{2}+{\left\|z\left(t\right)-\left(A+P\right)S\left(t\right)\right\|}_{2}^{2}+\varepsilon {\left\|S\left(t\right)\right\|}_{1},$$

where ε>0 is the regular parameter. The alternating descent method can be utilized to effectively solve Equation (17). Then, the above problem can be separated out two sub-optimization problems. 

First, fixing error term P, where *i* denotes the iteration indicator, the first sub-problem of Equation (17) can be formulated as:

(18) $$\begin{array}{l} \underset{S^{i}\left(t\right)}{\arg\min}{\left\|S^{i}\left(t\right)\right\|}_{1}\\ s.t.{\left\|z\left(t\right)-\left(A+P^{i-1}\right)S^{i}\left(t\right)\right\|}_{2}^{2}\leq \zeta \end{array}$$

where ζ is a presupposed threshold, the initial error term $P^{0}=0_{M\times Q}$. It is noticed that Equation (18) is completely consistent with the CS model. Hence, the $S^{i}\left(t\right)$ can be estimated by the greedy methods, here, we use the SOMP (Simultaneous Orthogonal Marching Pursuit) method. Then, substituting the estimated $S^{i}\left(t\right)$ into Equation (17). The second sub-problem is defined as:

(19) $$P^{i}=\underset{P}{\min}{\left\|P^{i-1}\right\|}_{F}^{2}+{\left\|z\left(t\right)-\left(A+P^{i-1}\right)S^{i}\left(t\right)\right\|}_{2}^{2}.$$

The close-form solution of the quadratic problem (19) can be obtained by making its first-order derivative equal to be zero, which can be expressed as:

(20) $$P^{i}=\left[z\left(t\right)-AS^{i}\left(t\right)\right]{\left(S^{i}\left(t\right)\right)}^{T}{\left[I+S^{i}\left(t\right){\left(S^{i}\left(t\right)\right)}^{T}\right]}^{-1},$$

where I denotes the identity matrix. The algorithm circularly implements between Equations (18) and (20). Its stop condition is ${\left\|S^{i}\left(t\right)-S^{i-1}\left(t\right)\right\|}_{2}\leq \varsigma$ or the maximum iteration number. Thus, all error terms $P_{k}$ and the SVs $a({\tilde{\theta }}_{k})$, k=0,1,⋯,K of the signals can be obtained according to the location of the $s_{k}\left(t\right)$ in $S^{i}\left(t\right)$. The ${\tilde{\theta }}_{k}$ is the angle corresponding to the estimated grid position where the *k*-th signal is located. Because the gain errors have been calibrated, the estimated phase errors of the *k*-th signal at *m*-th sensor can be expressed as:

(21) $$\delta \varphi_{k,m}=-j\times \left(P_{k,m}/A_{k,m}\right)\;m=1,2,\cdots ,M.$$

Finally, the phase error of the *m*-th sensor is given as:

(22) $$\delta \varphi_{m}=\frac{\sum_{k=0}^{K}\delta \varphi_{k,m}}{K+1}m=1,2,\cdots ,M.$$

Therefore, the compensation matrix is:

(23) $$E=\mathrm{diag}\left\{\frac{1}{\delta g_{m}}e^{-j\delta \varphi_{m}}\right\}m=1,2,\cdots ,M.$$

#### 3.2.3. The Estimation of Signal Number

In the above method, the sparsity of the signals should be previously estimated. For decades, various estimation methods of source number have been proposed, such as, the MDL [23], the AIC [24], and the GDE [25]. Among these type methods, the GDE can work efficiently in the situations of low SNR and measured data. Therefore, the GDE is utilized here to estimate the number of the signals. Construct a new covariance matrix, which can be written as:

(24) $$R_{c}=\left[\begin{array}{cc} {\hat{R}}_{1} & r\\ r^{H} & {\hat{R}}_{MM} \end{array}\right],$$

in which ${\hat{R}}_{1}$ is a front principal submatrix of $\hat{R}$ with dimension of (M−1)×(M−1), r is an (M−1) dimension vector at the last column of $\hat{R}$ without last element, ${\hat{R}}_{MM}$ denotes the element at the last row and column of $\hat{R}$. Now, a crucial transformation matrix $T_{c}$ can be constructed as:

(25) $$T_{c}=\left[\begin{array}{cc} U_{1} & 0\\ 0^{T} & 1 \end{array}\right],$$

where $U_{1}$ indicates an (M−1)×(M−1) unitary matrix, it can be obtained by eigen-decomposing the submatrix ${\hat{R}}_{1}$. The corresponding diagonal matrix $\Lambda_{c}$ is given as:

(26) $$\Lambda_{c}=\mathrm{diag}\left\{{\bar{\lambda }}_{1},{\bar{\lambda }}_{2},\cdots ,{\bar{\lambda }}_{M-1}\right\},$$

where ${\bar{\lambda }}_{1}\geq {\bar{\lambda }}_{2}\geq \cdots \geq {\bar{\lambda }}_{M-1}$. Then, the transformed covariance matrix as follows:

(27) $$T_{c}^{H}R_{c}T_{c}=\left[\begin{array}{cc} U_{1}^{H}{\hat{R}}_{1}U_{1} & U_{1}^{H}r\\ r^{H}U & {\hat{R}}_{MM} \end{array}\right]=\left[\begin{array}{cc} \Lambda_{c} & \rho \\ \rho^{H} & {\hat{R}}_{MM} \end{array}\right]=\left[\begin{array}{ccccc} {\bar{\lambda }}_{1} & 0 & \cdots & 0 & \rho_{1}\\ 0 & {\bar{\lambda }}_{2} & \cdots & 0 & \rho_{2}\\ \vdots & & & \vdots & \vdots \\ 0 & 0 & \cdots & {\bar{\lambda }}_{M-1} & \rho_{M-1}\\ \rho_{1}^{*} & \rho_{2}^{*} & \cdots & \rho_{M-1}^{*} & {\hat{R}}_{MM} \end{array}\right],$$

where $\rho =U_{1}^{H}r={\left[\rho_{1},\rho_{2},\cdots ,\rho_{M-1}\right]}^{T}$, the number of signals K+1 can be calculated by the GDE criterion:

(28) $$\varepsilon \left(n\right)=\left|\rho_{n}\right|-\frac{\tau }{M-1}\sum_{m=1}^{M-1}\left|\rho_{m}\right|,n=1,2,\cdots ,L-2,$$

where τ∈[0,1] denotes a regular factor. The number of the positive value in ε(n) is the number of signals.

### 3.3. INC Matrix Reconstruction

Here, according to the definition of the INC matrix proposed by Gu [15] and the angle sparsity of the interferences, the new INC matrix can be reconstructed as:

(29) $${\tilde{R}}_{I+N}=\sum_{k=1}^{K}{\tilde{\sigma }}_{k}^{2}a({\tilde{\theta }}_{k})a^{H}({\tilde{\theta }}_{k})+{\tilde{\sigma }}_{n}^{2}I,$$

where $a({\tilde{\theta }}_{k})$ is the SV of the *k*-th interference obtained from Section 3.2. ${\tilde{\sigma }}_{k}^{2}$ denotes the corresponding power value, which can be accurately estimated by solving the following quadratic optimization problem:

(30) $$\min{\left\|\Delta \upsilon \right\|}_{2}^{2}+{\left\|R_{v}-\tilde{A}\left(v+\Delta \upsilon \right)\right\|}_{2}^{2},$$

where $\upsilon ={\left[{\tilde{\sigma }}_{0}^{2},{\tilde{\sigma }}_{1}^{2},\cdots ,{\tilde{\sigma }}_{K}^{2}\right]}^{T}$ denotes the estimated powers corresponding to the SVs matrix $\tilde{A}$, Δυ indicates the difference between the estimated signal powers with the true values. $R_{v}=vec\left(E\hat{R}E^{H}\right)$, $\tilde{A}=\left[vec\left(a({\tilde{\theta }}_{0})a^{H}({\tilde{\theta }}_{0})\right),vec\left(a({\tilde{\theta }}_{1})a^{H}({\tilde{\theta }}_{1})\right),\cdots ,vec\left(a({\tilde{\theta }}_{K})a^{H}({\tilde{\theta }}_{K})\right)\right]$, where vec(·) is a vectorization operation, i.e., stacking all columns of a matrix on top of another, $E\hat{R}E^{H}$ indicates the SCM $\hat{R}$ is calibrated with the compensation matrix E.

To eliminate the noise uncertainties term, based on the TLS theory, the closed-form solution of Equation (30) can be expressed as:

(31) $$v=\left({\tilde{A}}^{H}\tilde{A}-\varsigma^{2}I\right){\tilde{A}}^{H}b,$$

where $\varsigma^{2}$ is the minimum eigenvalue of the matrix ${\left[\tilde{A}|b\right]}^{H}\left[\tilde{A}|b\right]$, $b=vec\left(E\hat{R}E^{H}-{\tilde{\sigma }}_{n}^{2}I\right)$. Now, the new INC matrix can be obtained according to Equation (29).

The proposed adaptive weight vector is:

(32) $$w_{0}=\frac{{\tilde{R}}_{i+n}^{-1}a\left({\tilde{\theta }}_{0}\right)}{a{\left({\tilde{\theta }}_{0}\right)}^{H}{\tilde{R}}_{i+n}a\left({\tilde{\theta }}_{0}\right)},$$

where ${\tilde{\theta }}_{0}$ is the estimated direction of the SOI from Section 3.2.

### 3.4. Algorithm Procedure

The proposed algorithm is summarized in Algorithm 1:
**Algorithm 1** The proposed robust adaptive beamformer**Step 1.** Estimate the sensor gain errors by Equation (16).**Step 2.** Estimate the number of signals by Equation (28).**Step 3.** The gain errors should be calibrated beforehand. When the iteration is terminated, calculate the directions of arrival (DOAs) of the signals and the phase errors of the sensors by (18) and (22), respectively. The signal DOAs can be obtained by finding the peak in the $S^{i}\left(t\right)$.**Step 4.** Calculate the calculation matrix using (23).**Step 5.** Estimate interference powers by Equation (31) after compensating the gain and phase errors.**Step 6.** Calculate the INC matrix with Equation (29).**Step 7.** The weight vector is calculated by (32).**Step 8.** The output signal after RAB processing can be obtained by (5).

## 4. Simulations

In this section, considering uniform linear array is composed of 10 omni-directional sensors spacing at half-wavelength. The gain errors ${\left\{\delta g_{m}\right\}}_{m=1}^{M}$ and the phase errors ${\left\{\delta \varphi_{m}\right\}}_{m=1}^{M}$ of the array are respectively generated by:

(33) $$\delta g_{m}=1+\sqrt{12}\delta_{g}\beta_{m},\;\delta \varphi_{m}=\sqrt{12}\delta_{\varphi }\eta_{m},$$

where $\beta_{m}$ and $\eta_{m}$ denote the independent and identically distributed random variables that are uniformly distributed at [−0.5,0.5], $\delta_{g}$ and $\delta_{\varphi }$ indicate the standard deviations of $\delta g_{m}$ and $\delta \varphi_{m}$, respectively. One SOI and two interference sources are assumed from directions 0°, −25°, 35°, respectively. In each sensor, the interference-to-noise ratio (INR) is set as 30 dB. The additive noise is spatially white Gaussian process. The norm direction of the SOI is equal to 2°, i.e., there is a 2° pointing error between the true and norm directions of the SOI. The random gain-phase errors and noise change from run to run while the other parameters are fixed. In the experiments below, $\delta_{g}=0.2$ and $\delta_{\varphi }=10{}^{\circ}$, the number of Monte Carlo trials is 300 for every scenario. 

The performance of the proposed algorithm is compared to the RC beamformer (RCB) [5], where the uncertainty parameter ε=3.25 is chosen; error compensation-based beamformer (ECBB) [18]; reconstruction-based beamformer (RBB) [13], the Gu’s beamformer [12], and the expanded RC beamformer (ERCB), where ε=0.6 is commended in [19]. For the RBB’s and Gu’s beamformers, the angular sector of the SOI is supposed to be $\Theta_{S}=\left[-5{}^{\circ},5{}^{\circ}\right]$, the corresponding complement sector is $\Theta_{I}=\left[-90{}^{\circ},5{}^{\circ}\right)\cup \left(5{}^{\circ},90{}^{\circ}\right]$, the integral interval is 1°. The maximum iterations number is 100 for the proposed beamformer.

### 4.1. Performance Analysis of the Gain and Phase Errors

**Figure 1:**
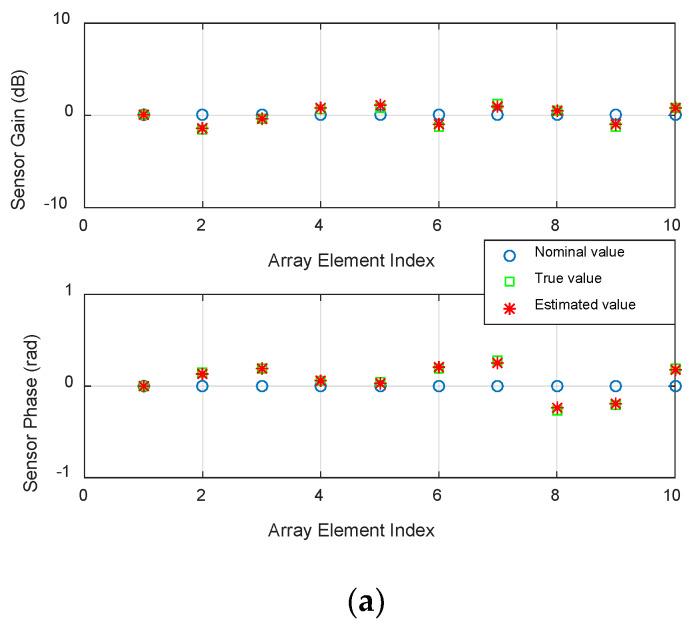

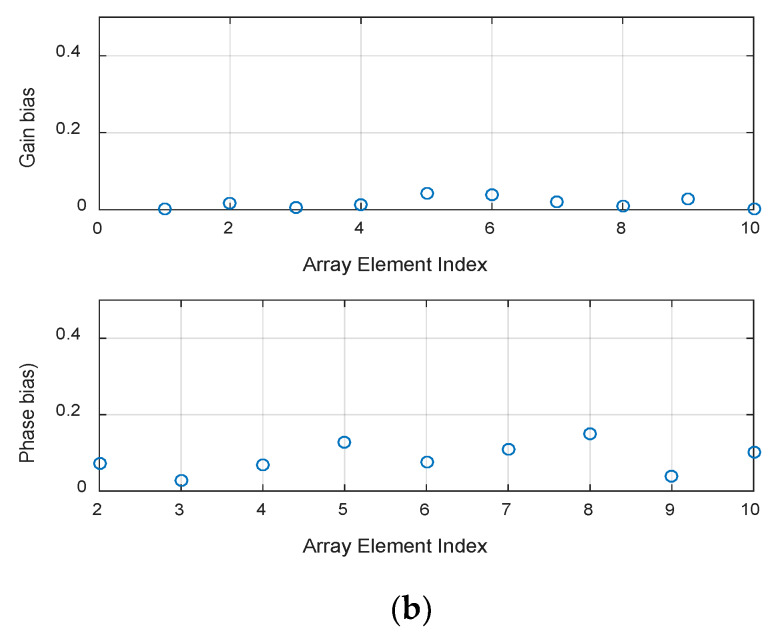
The estimated performance of the gain and phase errors: (**a**) The estimated results compared to the true values; (**b**) The estimated bias.

In the first test, the calibration performances of the gain and phase errors of the sensors are analyzed. Let one signal be incident on the array, the SNR is set as 20 dB. The DOA of the signal is 10°, the number of snapshots is 20. The estimated bias is defined as:

(34) $$bias=\frac{1}{T}\sum_{t=1}^{T}\left(\left|v_{t}-{\bar{v}}_{t}\right|/v_{t}\right),$$

where T is the number of snapshots, $v_{t}$ and ${\bar{v}}_{t}$ respectively denote the true values and the estimated ones. From Figure 1, it can be clearly seen that the estimated results of our algorithm is very closed to the true ones and has high accuracy.

### 4.2. Beampatterns Analysis of the Sparse Array

**Figure 2:**
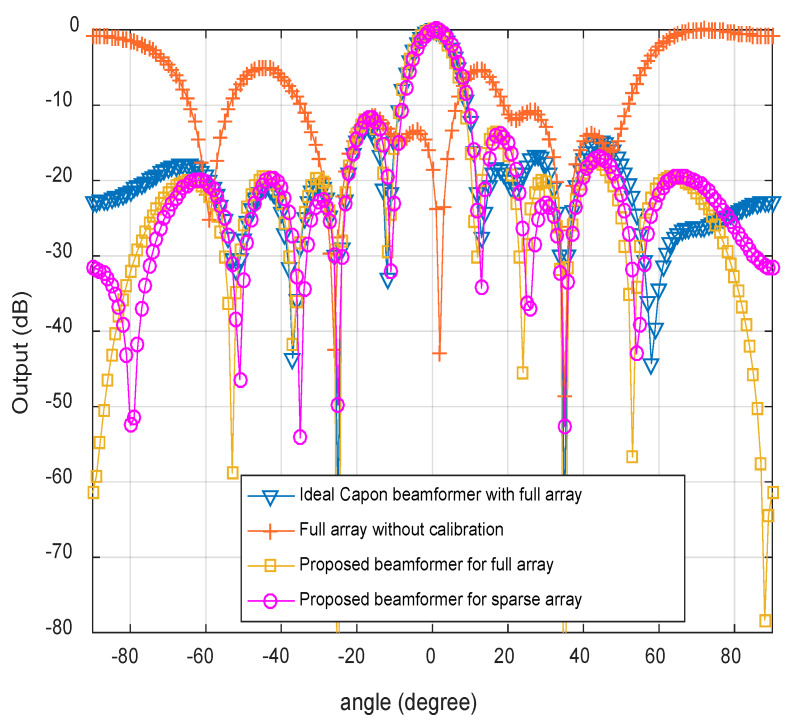
Beampatterns comparison.

The normalized beampatterns of the different conditions are shown in Figure 2 with SNR = 20 dB and snapshots *T* = 100. Here, we mainly compare the following four cases: full array without calibration, the proposed beamformer for full array, the proposed beamformer for sparse array (randomly selected 8 sparse elements, such as 1,3,4,5,7,8,9,10), the ideal Capon beamformer with full array. By contrast, we can know that the beampatterns of the proposed beamformer is very close to the ideal beamformer while the first side-lobe increases 3 dB. In addition, the proposed beamformer can effectively solve the problem of sparse array, which mainly owes to the joint application of CS and sparse reconstruction methods.

### 4.3. Output SINR Against SNR

**Figure 3:**
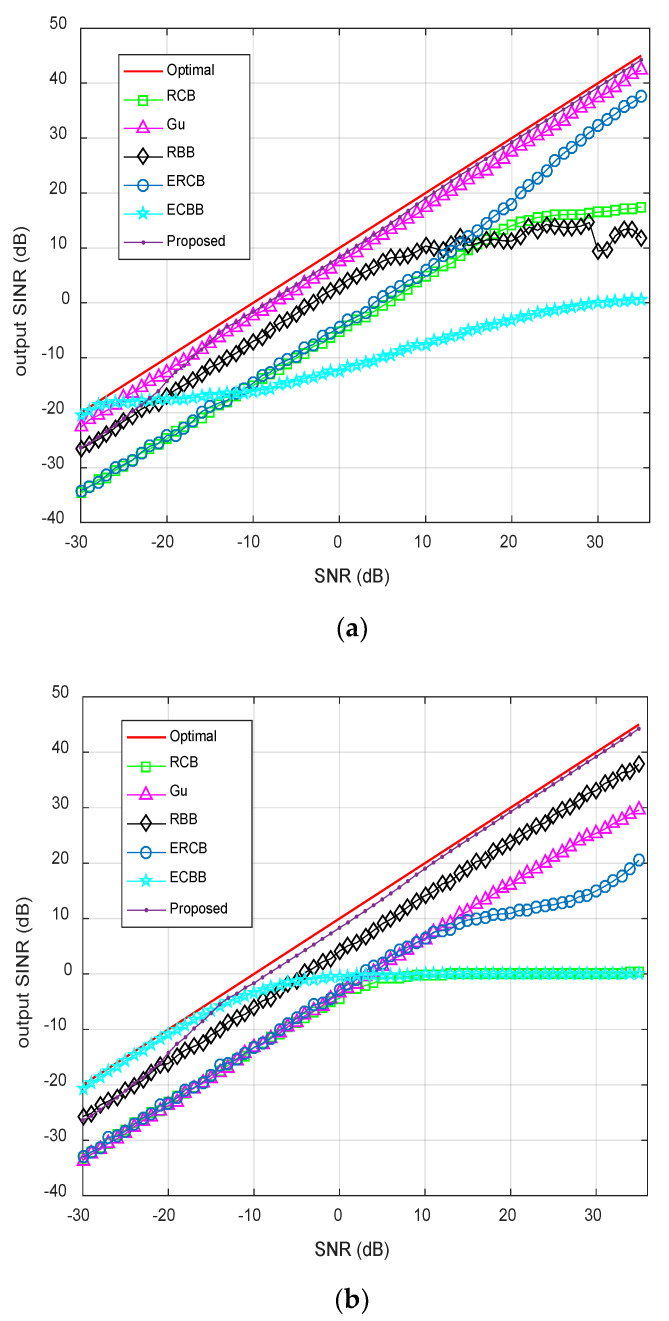
Output SINR against SNR with snapshots fixed at 50: (**a**) Incoherent sources; (**b**) Coherent sources.

Assume that the signals are incoherent sources when signal frequencies are different between each other, i.e., $f_{i}\neq f_{j}\left\{i\neq j\right\}\in \left[0,1,\cdots ,K\right]$. On the contrary, the signals are considered to be coherent sources. In this experiment, the output SINR performance versus the input SNR is investigated. The SNR varies from −30 to 35 dB. The number of snapshots is fixed to be 50. Figure 3a shows the comparison result of the output SINR in the situation of the incoherent signals. It is found that the performance of the proposed beamformer is very close to the optimal value in a wide range of input SNR. It can be seen from Figure 3b, the output SINR of the proposed algorithm has the same performance as the incoherent case. In the coherent situation, the proposed beamformer has at least 4 dB improvement over the other tested beamformers when the input SNR is higher than −5 dB. The output SINRs of Gu’s and RCB’s beamformers are severely degraded, this is mainly because the Capon technology cannot effectively distinguish coherent sources.

### 4.4. Output SINR Against the Number of Shapshots

**Figure 4:**
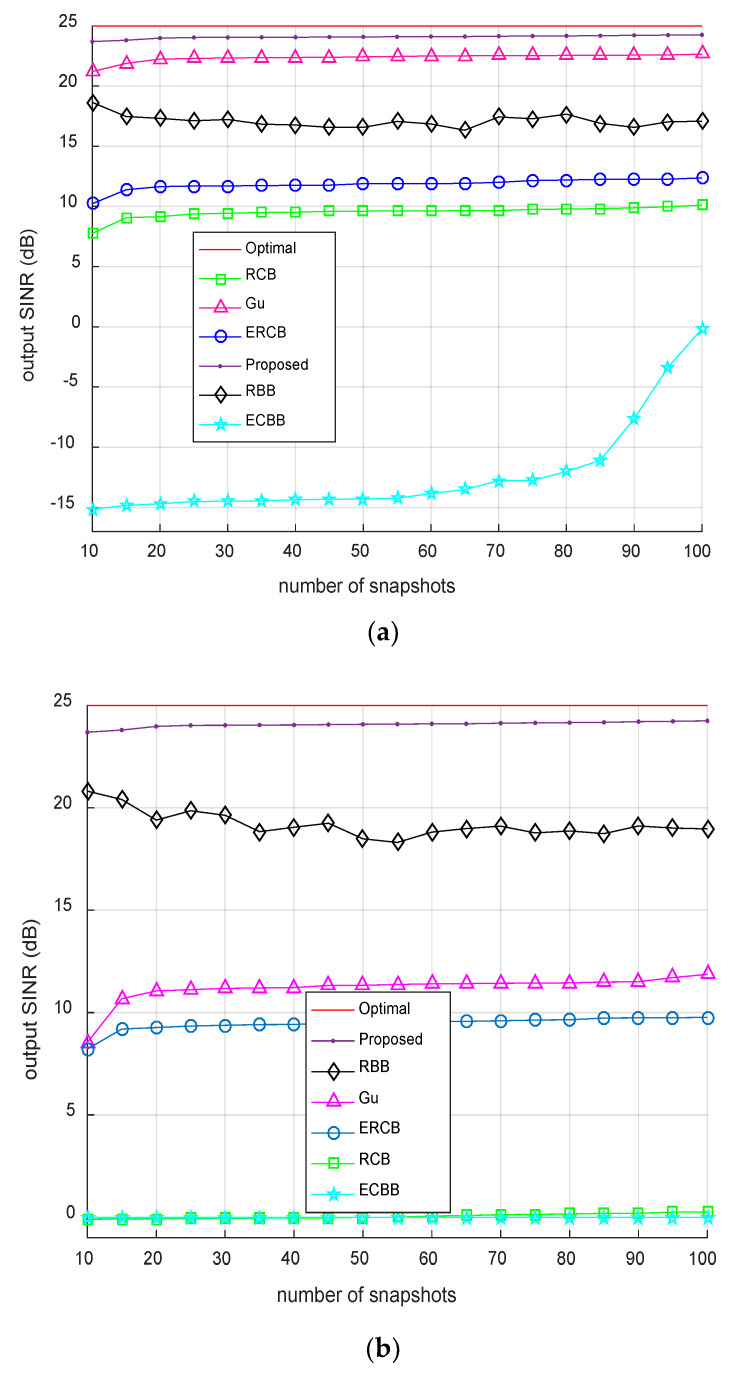
Output SINR against the number of snapshots with SNR fixed at 15 dB: (**a**) Incoherent sources; (**b**) Coherent sources.

In this test, the output SINR against the number of snapshots is investigated. The number of snapshots changes from 10 to 100. The input SNR is fixed to be 15 dB. It can be seen from Figure 4 that the convergence rates of the proposed beamformer are almost the same in the coherent and incoherent cases. Compared to the optimal beamforming, the output SINRs are only 0.7–1.3 dB loss. The proposed beamformer has much better performance than the other beamformers when the number of snapshots varies from 10 to 100, especially for the coherent sources.

## 5. Validation of the Measured Data

As shown above, the robustness of the proposed algorithm has been verified by the various simulations. In the practical system, robust adaptive beamformer is usually applied to suppress the side-lobe interference, while the gain and phase errors of the sensors can seriously degrade the mitigation performance. Hence, we focus on further analyzing its robustness with the measured HFSWR data in this section. As stated in [8,9,10], there are two issues that have a significant influence on the output SINR of the RAB. The first one is the interference suppression performance; the second one is the ability of preventing target self-cancellation. In the following, we will explore the performance of the proposed algorithm aiming at the above problems.

### 5.1. Analysis of Estimation Accuracy of Interference and Noise

**Figure 5:**
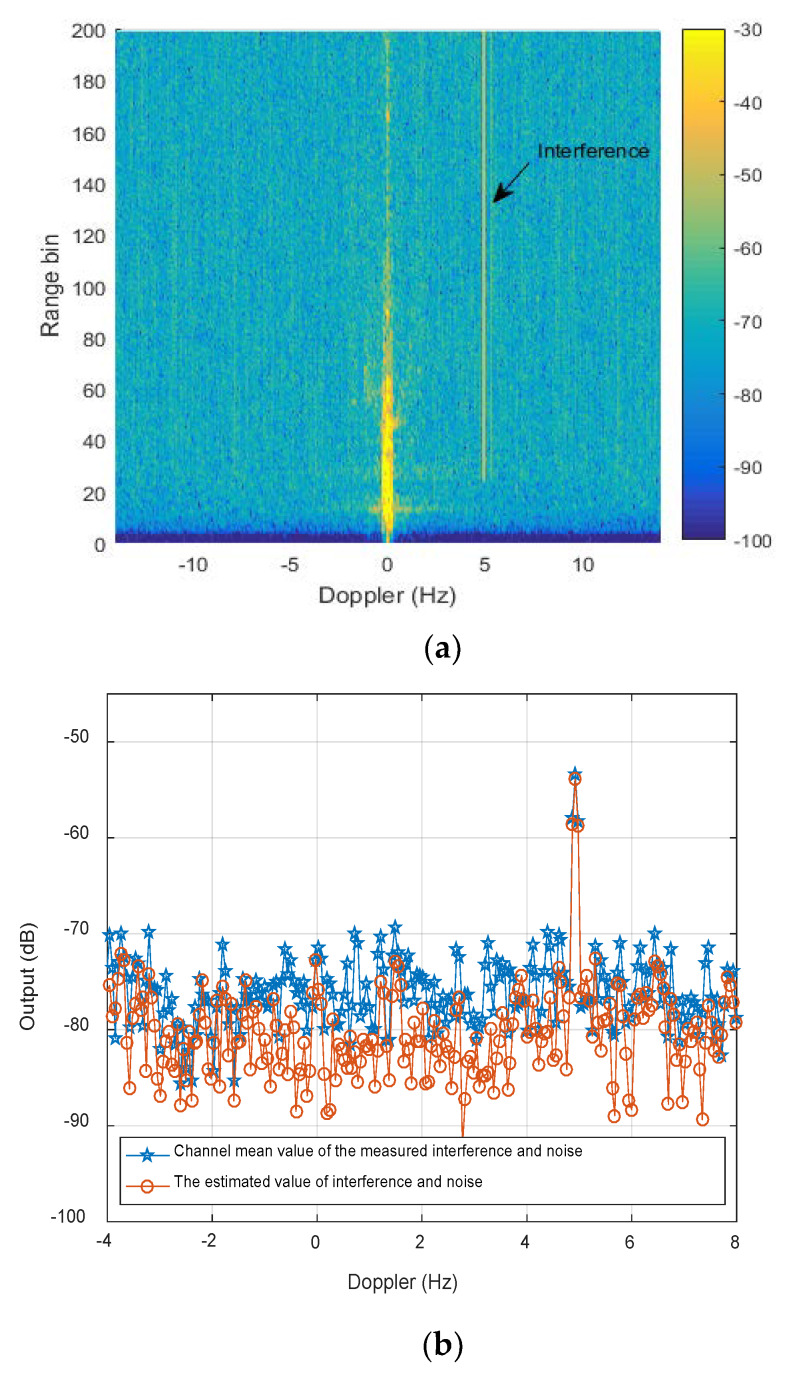
The estimated result with the measured HFSWR data: (**a**) Range-Doppler spectrum; (**b**) Doppler profile at the 100th range bin.

The measured range-Doppler spectrum of radio interference with HFSWR is shown in Figure 5a, which covers all the range bins above 25th bins. The number of snapshots is set as 20, the training samples are selected in range dimension of the interference area. From Equation (29), we can know, the reconstruction effectiveness of the INC matrix depends on the estimation precision of interference and noise. In this test, we determine the validity of the proposed Equation (30). In Figure 5b, the blue line expresses the statistical average of all channel data at the 100th range bin, the normalized interference power is about −53 dB, the noise power changes from −85 to −70 dB; the red line is the correspondingly estimated value based on Equation (30). It can be seen from Figure 5b, the estimated interference power equal to the measured value, two kinds of noise level are nearly equal. This shows the proposed reconstruction method of the INC matrix is still effective for the measured gain and phase errors.

### 5.2. Performance Analysis for Interference Mitigation and Precenting Target Self-Cancellation

**Figure 6:**
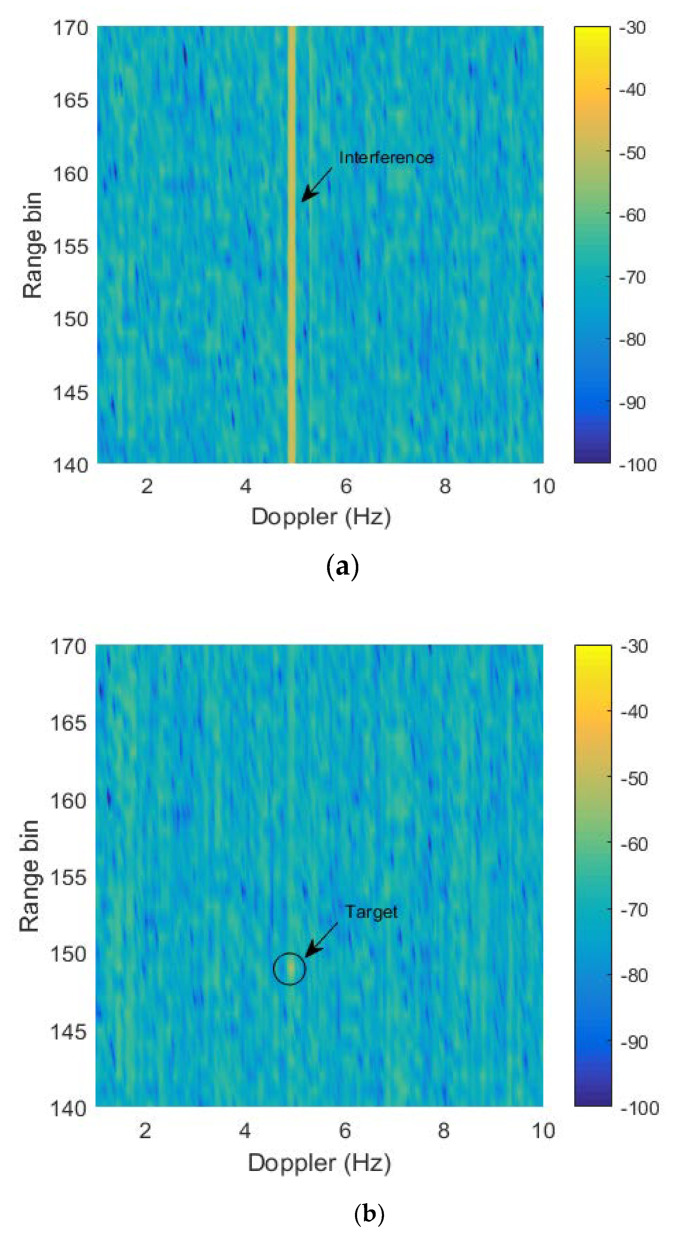

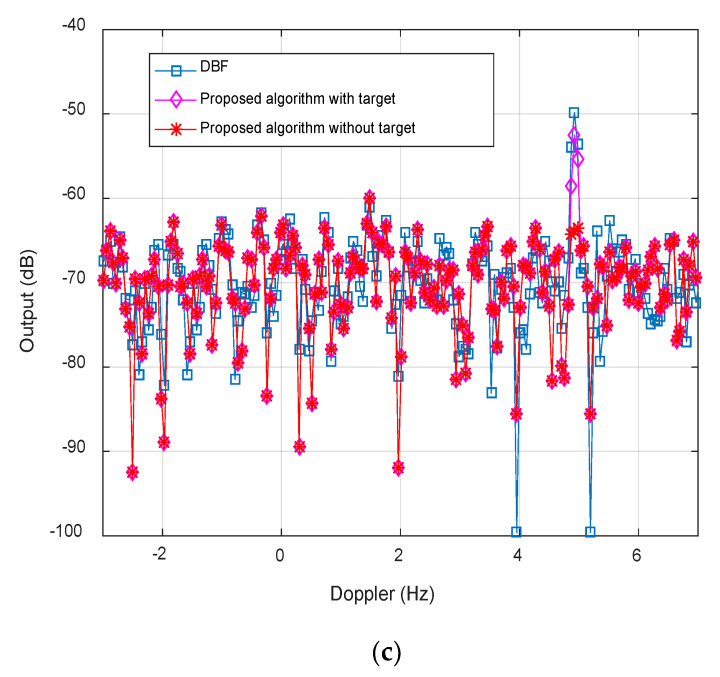
The cancellation results for radio interference: (**a**) Range-Doppler spectrum before suppression; (**b**) Range-Doppler spectrum after suppression; (**c**) Doppler profile after suppression.

This section quantitatively analyzes the abilities of interference suppression and preventing target self-cancellation of the proposed algorithm. Therefore, a simulated target with the same Doppler is injected into the interference. A locality enlarged spectrum of Figure 5a in the injected target position is shown in Figure 6a, the pointing error is still 2°. The detailed target and interference parameters are given in Table 1. In the corresponding area, the mitigation result with the proposed algorithm is shown in Figure 6b, which shows that the interference is significantly suppressed. Figure 6c displays a Doppler frequency profile at the 150th range bin after mitigation. It can be seen from Figure 6c, the targets only lose 0.3 dB while the interference is nearly mitigated 14 dB. In conclusion, the proposed method not only excellently suppresses interference, but well preserves target power.

## 6. Conclusions

In this paper, based on the sparse reconstruction method of the INC matrix, we propose a novel robust adaptive beamformer against sensor gain and phase errors for a full and sparse array. In order to eliminate the influence of sensor gain and phase errors, we derive an error model. On this basis, the SVs of signals and the errors can be accurately estimated. The accurate powers of the signals and noise can be obtained by solving a quadratic problem after the errors are calibrated. The simulation results demonstrate that when the input SNR is higher than −5 dB, the output SINR of the proposed method has at least 4 dB improvement than the other tested ones in the coherent case. The measured data show that the interference is mitigated about 14 dB, but the target only losses 0.3 dB. From the measured results, we can know that our algorithm not only can effectively suppress the real radio interference, but can significantly avoid the self-cancellation of the SOI. 

## Figures and Tables

**Table 1:** Target and interference parameters.

Target and Interference	Range Bin	Doppler	Angle	Input Power	Output Power
Interference	23th–200th	4.923 Hz	9°	−49.82 dB	−63.53 dB
Target1	150th	4.923 Hz	−20°	−52.20 dB	−52.51 dB
